# Double-Pulse Ultrasonic Welding of Carbon-Fiber-Reinforced Polyamide 66 Composite

**DOI:** 10.3390/polym14040714

**Published:** 2022-02-12

**Authors:** Qian Zhi, Yongbing Li, Peng Shu, Xinrong Tan, Caiwang Tan, Zhongxia Liu

**Affiliations:** 1 Hunan Engineering Research Center of Forming Technology and Damage Resistance Evaluation for High Efficiency Light Alloy Components, Hunan University of Science and Technology, Xiangtan 411201, China; zhiqianhnust@163.com (Q.Z.); shupeng2022@163.com (P.S.); 2 Shanghai Key Laboratory of Digital Manufacture for Thin-Walled Structure, Shanghai Jiao Tong University, Shanghai 200240, China; yongbinglee@sjtu.edu.cn; 3 Shandong Provincial Key Laboratory of Special Welding Technology, Harbin Institute of Technology at Weihai, Weihai 264209, China; tancaiwang@hitwh.edu.cn; 4 School of Physics and Engineering, Zhengzhou University, Zhengzhou 450052, China; liuzhongxia0@163.com

**Keywords:** ultrasonic welding, double pulse, temperature evolution, weld area, peak load

## Abstract

Ultrasonic welding of thermoplastics is widely applied in automobile and aerospace industries. Increasing the weld area and avoiding thermal decomposition are contradictory factors in improving strength of ultrasonically welded polymers. In this study, relations among the loss modulus of carbon-fiber-reinforced polyamide 66 composite (CF/PA 66), time for obtaining stable weld area, and time for CF/PA 66 decomposition are investigated systematically. Then, a double-pulse ultrasonic welding process (DPUW) is proposed, and the temperature evolutions, morphologies and structures of fractured surfaces, and tensile and fatigue properties of the DPUWed joints are measured and assessed. Experimental results show the optimal welding parameters for DPUW include a weld time of 2.1 s for the first pulse, a cooling time of 12 s, and a weld time of 1.5 s for the second pulse. The DPUW process enlarged the weld area while avoided decomposition of CF/PA 66 under appropriate welding parameters. Compared to the single-pulse welded joint, the peak load, weld area, and endurance limit of the DPUWed joint increased by about 15%, 23% and 59%, respectively. DPUW also decreases the variance in strengths of the joints.

## 1. Introduction

Polymer composites play an increasingly significant role in automobile and aerospace industries due to low density, high specific strength, and corrosion resistance [1]. Joining of thermoplastics in complex structures has become more and more prominent [2]. Ultrasonic welding (UW) is widely used in joining polymers because it is fast, economic, and easy for mass production [3,4]. This technique joins the separated parts by workpiece-to-workpiece friction and viscoelastic dissipation in the polymer (intermolecular friction) [5,6]. Energy directors are usually recommended to improve the energy efficiency during ultrasonic welding. However, the introduction of energy directors will bring problems, such as increased cost, easily restrained by the size and position of the workpieces. Consequently, ultrasonic welding of polymeric composites without energy directors has become a research hotpot in the manufacturing industry.

In ultrasonic welding of polymers without energy directors, the energy is not concentrated, and coulombic friction at the faying interface is less than that of the joint with energy directors. As a result, the workpieces need a longer time to melt, and the corresponding weld time is prolonged for UWed joints without energy directors [7,8,9]. However, thermoplastics produce viscoelastic dissipation under ultrasonic welding, which leads to an increase in workpiece temperature with the extension of weld time. When the temperature rises to above decomposition point of the polymer, thermal decomposition will occur and porous region will form, which severely deteriorates the weld quality. Sun [10] studied ultrasonically welded carbon-fiber-reinforced polyamide 6 and pointed out that when the weld energy was higher than the upper bound of 1100 J, the porosity increased in the weld zone and an over-weld was formed due to polymer overheating, which had a negative impact on weld quality. Zhang [11,12], Qiu [13], Li [14] and Zhi [15,16,17] also observed this similar phenomenon when joining other polymers by ultrasonic welding. To summarize from the published literatures, the weld area is insufficient and the weld ruptures through nugget when weld time is short, whereas thermal decomposition easily emerges in the workpiece with prolonged weld time, which are contradictory factors in enhancing joint strength. Therefore, there is an urgent need to develop methods to enlarge weld area but avoid thermal decomposition in the meantime for ultrasonically welded thermoplastic composites.

It is known that heat generation during ultrasonic welding depends on the loss modulus of polymer, while the loss modulus is closely related to temperature [2,4,18]. The ultrasonic welding process is fast, and thus, the loss modulus of the material is determined by the temperature prior to welding. Accordingly, weld quality can be improved by changing the temperature distribution of the adherends. Wang [19] proposed interfacial preheating treatment in ultrasonically welded polyamide 6 composites and found proper preheating conditions helped to concentrate more weld energy at the joining surface and effectively improved the joint quality. Zhi [18] preheated whole workpieces prior to ultrasonic welding, the optimum weld time shortened from 2.1 s to 1.5 s, and the degree of thermal decomposition decreased, because the lower loss modulus reduced heat generation in the workpiece. Though the loss modulus of the polymer at the beginning of welding could change by selecting proper preheating temperature and lowering the probability for thermal decomposition, this requires extra operations and has limited effect. Inspired by preheating treatment, a multipulse ultrasonic welding method is proposed to improve the weld quality while avoiding thermal decomposition. The residual heat from the last pulse preheats the workpieces before the application of another pulse. Heat is mainly generated at the faying interface at initial stage of welding [20]. After a short period of welding, materials at the faying interface melt first, and the temperature is higher than that in the middle of the workpiece. After cooling for a while, another ultrasonic pulse is applied on the exact same location of the existing weld to extend the weld size continuously. The temperature in the middle of the workpiece is maintained below the decomposition temperature of the material by adjusting the weld time and cooling time between two ultrasonic vibrations. Thus, weld quality will be improved by increasing energy dissipation at the faying interface while inhibiting thermal decomposition. Since the multipulse ultrasonic welding process has potential to improve weld quality of thermoplastic composites, it is imperative to investigate the mechanism of multipulse ultrasonic welding.

The present study was undertaken to develop a method of double-pulse ultrasonic welding (DPUW) for improving the weld quality of carbon-fiber-reinforced polyamide 66. The mechanism of DPUW is evaluated thoroughly by analyzing the relations among the loss modulus of the material, time for obtaining stable weld area, and time for decomposition occurrence. The temperature evolution, peak load, and fatigue properties of the DPUWed joint are assessed systematically.

## 2. Materials and Experimental Procedure

The material used in this research was carbon-fiber-reinforced polyamide 66 composite with 30 wt% fiber (CF/PA 66) (Tianfu Co., Ltd, Shanghai, China), where the carbon fiber was about 2 mm in length with a diameter of 7 μm. All the coupons were injection-molded with dimensions of 132 × 38 × 4.0 mm^3^, and the injected CF/PA 66 exhibited a tensile strength of 99.2 MPa. During the ultrasonic welding process, a KZH 2026 welder with a nominal power of 2.6 kW, nominal frequency of 20 kHz, amplitude of 25 μm, and circular aluminum horn with a diameter of 18 mm were utilized. Time mode was chosen as welding mode.

**Figure 1:**
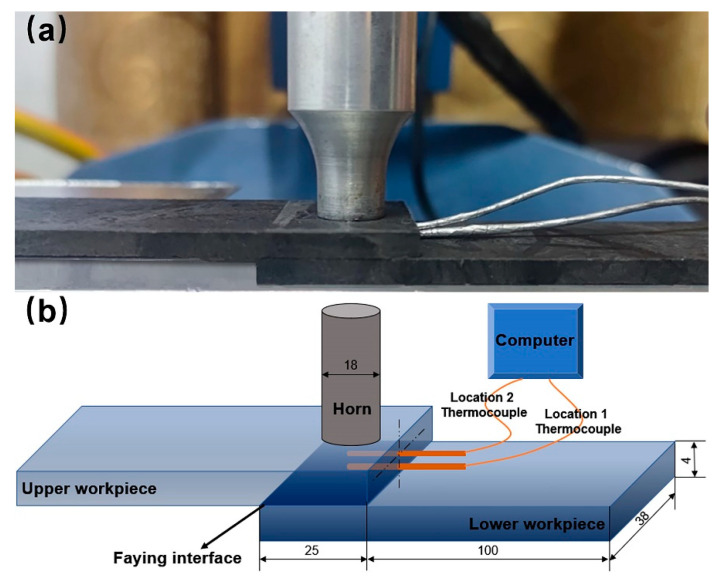
(**a**) Real figure and (**b**) Schematic of the temperature measurement during ultrasonic welding. (Dimensions in mm).

To analyze the weld formation during ultrasonic welding, temperature evolutions near the faying interface and in the middle of upper workpiece were measured. Figure 1a shows the real figure, and Figure 1b illustrates the sketch of temperature measurement during ultrasonic welding. As shown, two small holes with a diameter of 1.0 mm were drilled at 0.2 mm from the bottom surface and middle of the upper workpiece. Two K-type thermocouples were embedded into the two small holes and secured with epoxy compound to fix the thermocouples.

Loss modulus of CF/PA 66 was carried out by dynamic mechanical analysis (DMA, 242E, Netzsch, Selb, Germany). Specimens with dimensions of 38 × 8.5 × 4.0 mm^3^ were subjected to three-point bending with a span length of 20 mm. An oscillating force (≤4 N) was applied to give constant deflection amplitude of 30 μm. Measurements were conducted in the temperature range of 23–200 °C with a heating rate of 2 °C/min and fixed frequencies (1, 2, 5, 10 Hz). The loss modulus at 20 kHz was extrapolated by the time temperature superposition. The cross-sectioned morphologies of the welds were characterized by scanning electron microscopy (SEM, JSM 6700F, JEOL, Tokyo, Japan). All samples were sputter-coated with platinum for 50 s before SEM to induce conductivity. Chemical structures of the polymer were characterized using Fourier transform infrared spectroscopy (FTIR) (Perkin Elmer, Waltham, USA) Perkin Elmer Spectrum One. Pellets made of CF/PA 66 powder were diluted in KBr, and FTIR spectra were recorded between 4000 and 400 cm^−1^. The powder was obtained by grinding in an agate mortar with a pestle under an infrared lamp. Shear tensile and fatigue tests were performed on an MTS 810 servohydraulic testing machine. To minimize the bending stresses inherent in the testing of single-lap weld specimens, filler plates were attached to both ends of the specimen using masking tape to accommodate the sample offset. The stroke rate was 2.0 mm/min for the tensile test, while the cyclic frequency was 30 Hz, and sinusoidal load cycles with a load ratio R = 0.1 were applied in fatigue test. Three replicates were tested, and the average values were reported for each welding condition.

## 3. Results and Discussion

### 3.1. Temperature Evolution in Ultrasonic Welding

**Figure 2:**
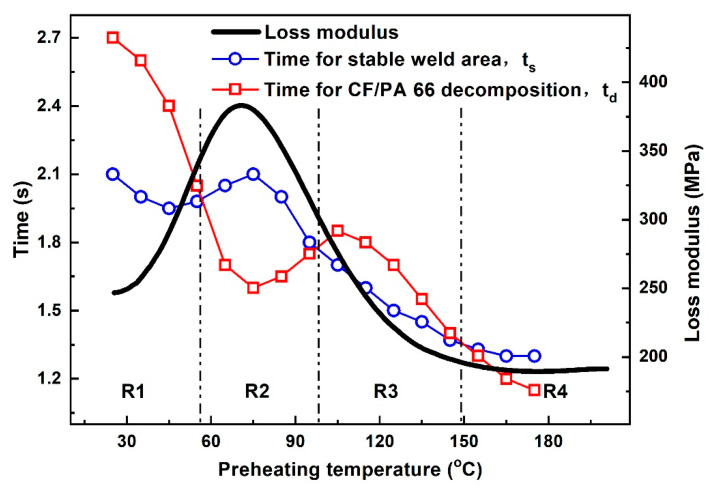
Relations among preheating temperature, loss modulus, t_s_, and t_d_.

There are two leading heating systems during ultrasonic welding of polymer composites: surface friction and viscoelastic dissipation. The loss modulus of CF/PA 66 composite is closely related to the condition of CF/PA 66, while the condition of CF/PA 66 rests on temperature. For ultrasonic welding process, the weld time only takes several seconds and the state transformation does not take place; therefore, the initial loss modulus at the very beginning of welding is identified as the loss modulus of CF/PA 66 during welding. The loss modulus of CF/PA 66 composite at 20 kHz (black curve) as a function of temperature is shown in Figure 2. The loss modulus of CF/PA 66 increases firstly and then decreases, and the peak value occurs at around 75 °C. In ultrasonic welding of polymers, the deficient weld area leads to weak joint strength. While prolonging weld time would extend the weld area or cause thermal decomposition of polymers [17,19], however, increasing weld area and polymer decomposition are contradictory in improving joint strength. Thus, two important parameters, i.e., t_s_ and t_d_, are also given in Figure 2 (according to our previous studies [16,18], weld pressure of 0.15 MPa is optimum for welding; thus, the following experiments are all conducted at a weld pressure of 0.15 MPa). t_d_ is defined as the time when the workpiece starts to decompose and forms a porous area, while t_s_ is the time when the joint obtains stable weld area. Numerous studies have shown that the weld area of the joints increases rapidly with weld time, and the weld area remains stable at a certain weld time [2,4,8,16]. This particular weld time is defined as t_s_, and the corresponding joint exhibits the highest strength. Further increasing the weld time, thermal decomposition emerges in the joint and the weld strength drops.

t_s_ and t_d_ are the results of extensive experiments (from preheating temperatures of 25 °C to 175 °C) [18]. Generally, t_s_ and t_d_ decrease as the preheating temperature rises, and the peak values occur at approximately 75 °C, which is likely correlated with the preheating temperature and corresponding loss modulus of the workpiece. The decreases in t_s_ and t_d_ are mainly because the preheating treatment provides energy for weld formation and workpiece decomposition. The peak and valley located at 75 °C are present because the maximum loss modulus of CF/PA 66 is at this temperature. The larger loss modulus leads to higher energy dissipation in the workpiece and relatively lower heat generation at the faying interface. Therefore, time for obtaining stable weld area prolongs, and the time for thermal decomposition shortens.

Based on the curves of time for stable weld area and time for CF/PA 66 decomposition in Figure 2, three intersections are observed, and the whole range is divided into four regions, denoted as R1, R2, R3, and R4. In R1 and R3, t_s_ is smaller than t_d_, implying the joint achieves its maximum weld area before thermal decomposition occurs in the joint. Accordingly, R1 and R3 are the appropriate regions, and t_s_ is the proper weld time for joints with preheating temperatures ranging from 25–55 °C and from 95–145 °C. In regions R2 and R4, t_s_ is larger than t_d_, indicating the composite decomposes before the joint obtains stable weld area. Assuming the workpiece does not decompose, then the weld area of the joint is smaller, and the joint strength is lower [18]. Consequently, R2 and R4 regions should be avoided in selecting the preheating temperature for ultrasonically welded CF/PA 66 without energy directors.

**Figure 3:**
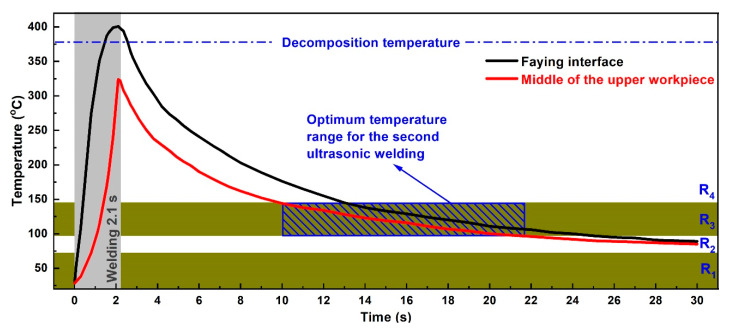
Temperature evolutions at the faying interface and middle of upper workpiece for joint welded with weld time of 2.1 s and weld pressure of 0.15 MPa.

**Figure 4:**
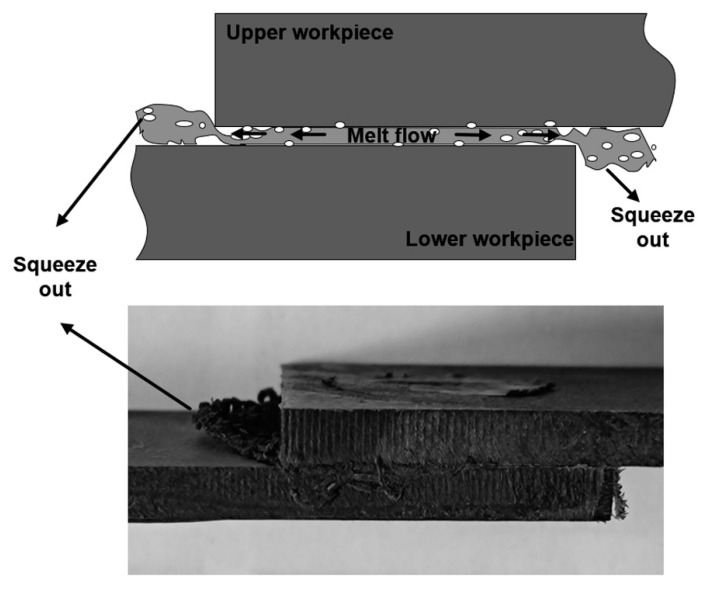
Morphology of the as-welded joint.

In practical production, ultrasonic welding usually operates at room temperature, and the ambient temperature of 25 °C, which lies in R1, is selected for the first ultrasonic vibration. As shown in Figure 2, the corresponding proper weld time for ultrasonic welding of CF/PA 66 composite at 25 °C is 2.1 s and is regarded as the optimum weld time for the first pulse. Once the weld time for the first ultrasonic vibration is determined, the cooling interval between two pulses needs to be ascertained. Since the preheating temperature is closely related with the weld quality, temperature before application of the second pulse should be in the ranges of R1 and R3. Transient temperature evolutions at the joining interface and middle of upper workpiece during ultrasonic welding of CF/PA 66 composite at 25 °C are measured and recorded, as shown in Figure 3. The temperatures at both positions show similar tendencies, which increase during welding and then decrease after the vibration stops. According to the theory above, R1 and R3 are the appropriate regions for preheating. Notice that R1 is in the range of 25–55 °C, and it takes a long time for the joint to cool down, which is not applicable in terms of efficiency. Accordingly, R3 region, i.e., 95–145 °C, is the suitable initial temperature for the second pulse. Selection of preheating temperature for ultrasonically welded CF/PA 66 is mainly dependent on temperature in the middle of the upper workpiece, because most of the decomposed materials at the faying interface are squeezed out and flow bilaterally under weld pressure, as shown in Figure 4. Therefore, the porous area would not be formed, as the temperature at the faying interface is higher than the decomposition temperature of CF/PA 66. While the materials in the workpiece cannot flow, if the temperature in the workpiece is above the decomposition temperature, the workpiece will decompose and result in pores and porous areas. Thus, the joint strength would be deteriorated.

It takes about 8 s for the middle of the upper workpiece to cool to R3 region, as shown in Figure 3. Considering the temperature gradient in the upper workpiece and ensuring the temperature of the whole upper workpiece lies in the range of R3, an interval time of 12 s between two ultrasonic pulses is adopted. Referring to Figure 2 and Figure 3, a weld time of 1.5 s is selected as the weld time for the second vibration. Therefore, the welding parameters for DPUW include a weld time of 2.1 s for the first ultrasonic pulse and a cooling time of 12 s, followed by a weld time of 1.5 s for the second vibration.

**Figure 5:**
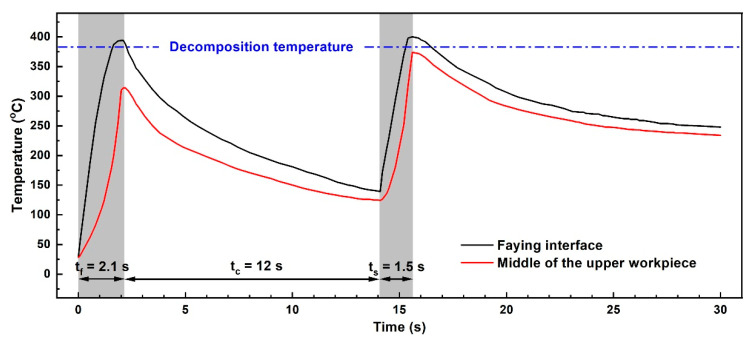
Temperature histories during double-pulse ultrasonic welding of CF/PA 66 composite.

Temperature histories for DPUWed joint at the faying interface and middle of the upper workpiece are measured, and the results are displayed in Figure 5. The temperature in the middle of the upper workpiece is always lower than that at the faying interface. It is also clear that the temperature in the middle of the upper workpiece during DPUW process is lower than the decomposition temperature of CF/PA 66 composite (i.e., 375 °C [21]), implying the composite does not decompose in the workpiece, which is in accordance with the design above.

### 3.2. Mechanical Property of the Joint

**Figure 6:**
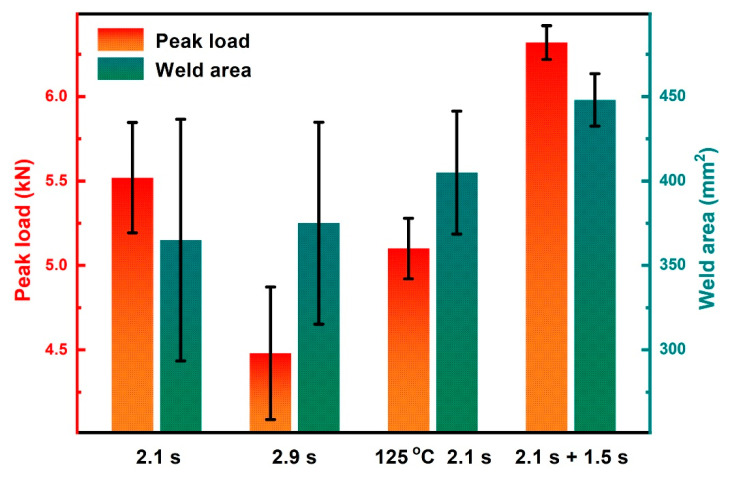
Peak loads and weld areas of joints welded with various welding parameters.

Figure 6 compares the peak loads of joints welded with different weld parameters, i.e., weld time of 2.1 s, 2.9 s, and 2.1 s, at a preheating temperature of 125 °C and DPUW. The joint with 2.1 s weld time shows a peak load of 5.52 kN and weld area of 365 mm^2^. After the applications of weld time for 2.9 s, preheating at 125 °C, and DPUW, the weld areas increase when compared to that of the joint welded with 2.1 s. Moreover, DPUWed joint exhibits increased peak load (higher by about 15%) and limited scatter, while the peak loads drop for the other two groups. These characteristics are probably related to the morphologies of the joints.

**Figure 7:**
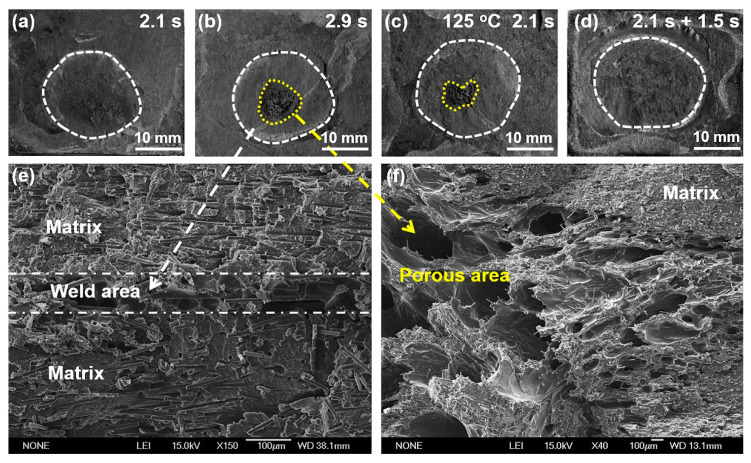
Morphologies of fractured surfaces: (**a**) 2.1 s weld time; (**b**) 2.9 s weld time; (**c**) 2.1 s weld time under preheating temperature of 125 °C; (**d**) DPUWed joint; cross-sectioned joint (**e**) weld area; and (**f**) porous area.

**Figure 8:**
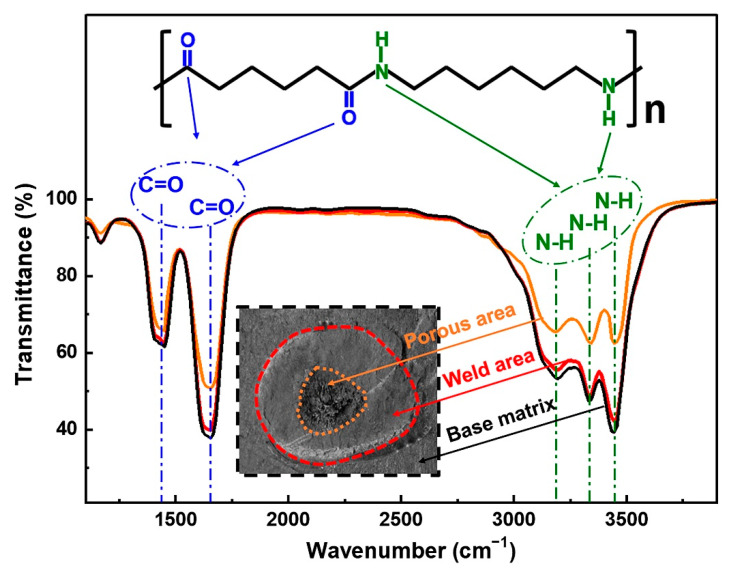
FTIR spectra of the matrix, weld area, and porous area.

Fractured surfaces of these joints are carefully examined and presented in Figure 7. The weld area is roughly circular in shape for the joint made with 2.1 s, and no obvious porous area is displayed at the faying interface, as shown in Figure 7a. Further prolonging the weld time to 2.9 s, the weld area enlarges slightly, and an obvious porous area occurs. SEM images of the cross-sectioned joint (as-welded) welded with 2.9 s are given in Figure 7e,f. Figure 8 shows the FTIR spectra of the matrix, weld area, and porous area. The morphology of weld area is similar to that of the matrix except for existence of random pores, which are probably formed due to ultrasonic cavitation or a small amount of polyamide decomposition [22]. The peak intensity of the weld area is also close to that of the matrix, indicating the workpiece only melts and solidifies during welding process, and no significant decomposition occurs. However, the quantity and size of pores grow significantly, and the microstructure of the porous area becomes loose compared with that of the weld area. Based on the FTIR results, the peaks’ intensities of C=O stretching at 1650 cm^−1^ and N-H stretching at around 3300 and 3400 cm^−1^ decrease primarily, which indicates that these groups fracture, and the polymer decomposes severely [23,24]. The decomposition of polyamide 66 releases volatile products such as NH_3_, CO_2_, CO, which results in a fragile porous area and deteriorates the joint strength [15,25].

Similar to the joint with 2.9 s weld time, a porous area shows at the faying interface for the preheated joint, and peak load of the joint decreases. It is seen that extending the weld time and preheating treatment cannot enlarge the weld area significantly and improve weld quality. The weld area of the DPUWed joint increases by about 23% compared with that of the joint welded for 2.1 s. This phenomenon indicates the double-pulse ultrasonic welding process indeed increases the energy dissipation at the faying interface. In addition, overheating does not emerge, and no porous area occurs in the joint due to controlling the temperature distribution. These results indicate the selection of DPUW parameters based on the temperature in upper workpiece is reasonable. It is worth mentioning that the double-pulse ultrasonic welding process not only improves the weld quality but also decreases the variance in peak load, which is beneficial for producing continuous solid joints. This result is likely correlated with the contact condition at the faying interface. It is difficult to guarantee absolute flatness of the workpiece during manufacturing. Thus, the contacts between workpieces differ at the initial stage of welding. Surfaces with higher asperities usually generate more heat and obtain intimate contact between workpieces. By contrast, heat generation becomes limited for joints with loose contact and results in insufficient welds. However, the asperities suffer from melting and solidifying after application of the first ultrasonic pulse, which results in a relatively flat faying interface; thus, the second ultrasonic energy can be evenly distributed at the faying interface. Hence, the stable weld seam is obtained with the DPUW process and the spread in peak load narrows. The specific mechanism for the decreased scatter in double-pulse ultrasonic welding process will be further studied in future research.

**Figure 9:**
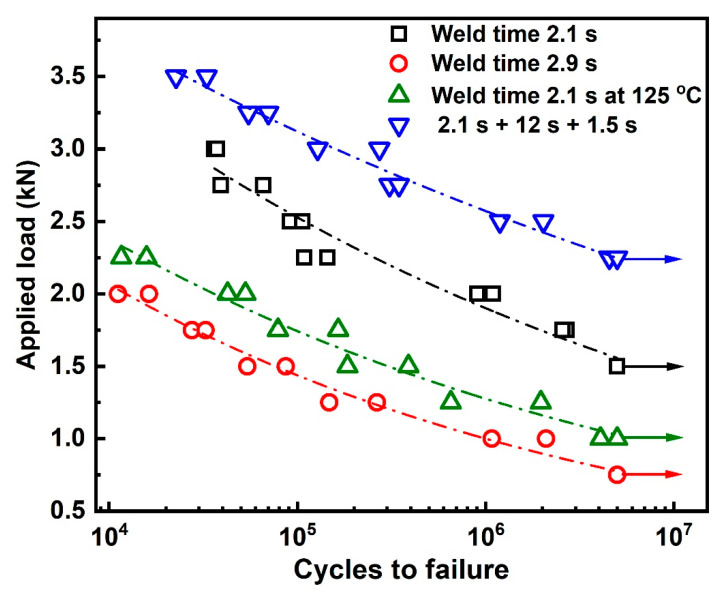
Fatigue life data for joints with various welding parameters.

The fatigue property of a joint is primarily important, because it inevitably suffers from complex and repeated mechanical loading during service. Figure 9 illustrates S–N (stress vs. number of cycles to failure) curves for joints welded with the abovementioned parameters. The specimen is defined to be running out when the loading cycle *N_f_* > 5 × 10^6^. A decreasing trend in fatigue lives with increasing mean stresses is observed under all weld conditions. A power law relation between the *S* and *N_f_* is assumed in line with Basquin equation and can be expressed as [26,27,28]:

*S* = *AN_f_^b^* (1)

where *S* is the stress, *N_f_* is the number of cycles to failure, and *A* and *b* are the fatigue strength coefficient and exponent, respectively. The approximated values of these coefficients, endurance limits, and correlation coefficients (R^2^) are summarized in Table 1. The correlation coefficients of R^2^ are close to 1, which shows good correspondence between tested and fitted results. The endurance limits of the joints mentioned above are 1.25 kN, 0.52 kN, 0.69 kN, and 1.99 kN. It is evident from Figure 9 and Table 1 that the fatigue property of DPUWed joints is significantly higher than those of preheated and single-pulse welded joints. This is similar to the results obtained from the tensile test. There are three possible reasons for the improvement in fatigue property of DPUWed joints: first, the weld strength of joint increases, as shown in Figure 6. Second, the residual heat from the first vibration preheats the workpieces before application of the second pulse, which reduces the temperature gradient between weld seam and matrix and thus decreases the residual stress in joint. Third, the insufficient weld caused by the rough workpiece is eliminated, and the stability of the joints is improved.

### 3.3. Exploration of DPUW Process Parameter

To validate the correctness of the above analysis and results, experiments with three process parameters with three levels are conducted. The process parameters are weld time of the first pulse (T_f_), cooling time (T_c_) and weld time of the second pulse (T_s_). Three levels of 1.5 s, 2.1 s, 2.7 s for T_f_; 6 s, 12 s, 28 s for T_c_; and 0.9 s, 1.5 s, 2.1 s for T_s_ are adopted. Parametric values for each experimental run and the average peak load, weld area, and existence of an obvious porous area are given in Table 2. Values of joint made with weld time of 2.1 s are also included in Table 2 as a reference. Referring to Table 2, porous areas occur in joints with T_f_ of 2.7 s irrespective of the cooling time and second weld time. This characteristic indicates that the weld time for the first ultrasonic pulse should be shorter than 2.7 s, and weld time of 1.5 s and 2.1 s are appropriate. It is seen from samples 12 to 18 that peak loads of joints with cooling intervals of 12 s are higher than those with cooling intervals of 6 s and 28 s. Combined with the temperature evolutions in Figure 3 and Figure 5, the temperatures in workpieces for joints with 12 s colling are located in R3, which is preferable for application of the second pulse. The highest peak load is obtained from sample 14 with an average of 6.32 kN, which is 15% higher than that of the reference joint.

**Figure 10:**
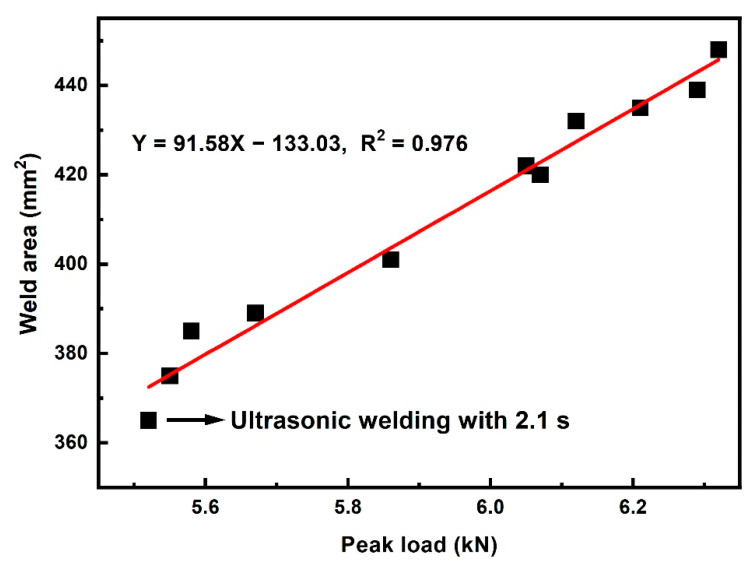
Relation between peak load and weld area for DPUWed joints.

Normally, the peak load of a joint is proportional to the weld area for joint without porous area [16,19,21,29]. However, whether this rule is applicable to double-pulse ultrasonic welding process still remains uncertain. Figure 10 shows the correlation between peak load and weld area of DPUWed joint with compact weld area. The red solid line in Figure 10 is the fitted curve. The correlation coefficient, R^2^, is 0.976, indicating the fitting is reliable. The relation can be described as:

Y = 91.58X − 133.03(2)

where Y is the weld area of DPUWed joint, and X is the peak load of the joint. As seen, the peak load is correlated linearly with weld area in the range of applied welding parameters. Therefore, as long as the faying surface of joint is compact (without pores), the peak load is directly proportional to weld area irrespective of the welding conditions.

## 4. Conclusions

Increasing the weld area and avoiding thermal decomposition are contradictory factors in improving strength of ultrasonically welded CF/PA 66. Ultrasonic welding of CF/PA 66 with different preheating temperatures was investigated thoroughly. The time for obtaining stable weld area and time for decomposition of CF/PA 66 decreased with increasing preheating temperature except for temperature around 75 °C. The occurrences of peak and valley values were mainly because the maximum loss modulus of CF/PA 66 was at this temperature. The proper temperature range for ultrasonic welding was R1 and R3, and the optimum temperature before application of the second ultrasonic pulse lay in the R3 region, i.e., 95–145 °C.

A method of double-pulse ultrasonic welding of CF/PA 66 is proposed based on the relations among loss modulus of CF/PA 66, time for obtaining stable weld area and time for material decomposition. The appropriate welding parameters for DPUW include a weld time of 2.1 s for the first pulse, 12 s cooling, and a weld time of 1.5 s for the second pulse. Results show that the DPUW process enlarged the weld area and avoided decomposition of CF/PA 66 in the meantime. Compared with welds made with prolonged weld time and preheat treatment, DPUWed weld exhibits higher peak load, larger weld area, more compact microstructure, and better fatigue property. The DPUW schedule not only improves the weld quality of ultrasonically weld CF/PA 66 but also decreases the spread in tensile and fatigue properties.

## Figures and Tables

**Table 1:** Fatigue S–N curve data for ultrasonically welded CF/PA66 composite.

Weld Condition	A	b	Endurance Limit Based on 5 × 10^6^ (kN)	R^2^
2.1 s	10.50	−0.12	1.25	0.94
2.9 s	8.81	−0.16	0.52	0.96
Preheated	8.27	−0.14	0.69	0.96
DPUW	8.2	−0.08	1.99	0.98

**Table 2:** Test data for DPUWed CF/PA66 composite.

Sample	T_f_ (s)	T_c_ (s)	T_s_ (s)	Peak Load (kN)	Weld Area (mm^2^)	Obvious Porous Area
1	1.5	6	0.9	5.55	375	N
2	1.5	6	1.5	5.3	388	Y
3	1.5	6	2.1	5.05	392	Y
4	1.5	12	0.9	5.67	389	N
5	1.5	12	1.5	6.05	422	N
6	1.5	12	2.1	6.18	428	N
7	1.5	28	0.9	5.86	401	N
8	1.5	28	1.5	6.12	432	N
9	1.5	28	2.1	5.41	441	Y
10	2.1	6	0.9	5.58	385	N
11	2.1	6	1.5	5.16	395	Y
12	2.1	6	2.1	4.82	407	Y
13	2.1	12	0.9	6.21	435	N
14	2.1	12	1.5	6.32	448	N
15	2.1	12	2.1	6.09	459	Y
16	2.1	28	0.9	6.07	420	N
17	2.1	28	1.5	6.29	439	N
18	2.1	28	2.1	5.28	455	Y
19	2.7	6	0.9	4.25	393	Y
20	2.7	6	1.5	3.82	398	Y
21	2.7	6	2.1	3.51	402	Y
22	2.7	12	0.9	5.45	386	Y
23	2.7	12	1.5	5.27	402	Y
24	2.7	12	2.1	5.04	407	Y
25	2.7	28	0.9	4.59	388	Y
26	2.7	28	1.5	4.35	397	Y
27	2.7	28	2.1	4.14	407	Y
Reference	2.1	/	/	5.52	365	N

## Data Availability

The data presented in this study are available on request from the corresponding author.
